# Cure of inguinal hernias with large preperitoneal prosthesis: Experience of 2,312 cases

**DOI:** 10.4103/0972-9941.27725

**Published:** 2006-09

**Authors:** J H Alexandre, J L Bouillot, P Dupin, K Aouad, J P Bethoux

**Affiliations:** Department of General and Digestive Surgery, Hotel Dieu University Hospital Place du Parvis de Notre Dame, Paris, France

**Keywords:** Femoral hernias, giant hernias, inguinal hernias, preperitoneal mesh, recurrent hernias

## Abstract

It is clear that the recurrence rates after nonprosthetic methods for the repair of inguinal hernias, like McVay, Bassini or Shouldice techniques, are high (6–10%). Since 20 years, we are convinced, in the GREPA-EHS group, about the advantages of the use of a prosthetic mesh in majority of patients for repairs of primary or recurrent inguinal hernias and incisional hernias. We describe our typical technique for the cure of all inguinal hernias. We place a large supple mesh, by open inguinal route, posterior to the transversalis fascia and anterior to the peritoneum. We have made a double modification in the initial technique of Rives - the use of a very large unsplit prosthesis (15 × 17 cm) and the parietalization of the spermatic cord helped by a wide opening of the Fruchaud's orifice by diversion of the epigastric vessels. The positioning of the mesh is about the same as in the TEP technique but with the advantages of reduction in the vital laparoscopic risks and reinforcement of the wall by a short tension-free McVay technique.

For this prospective study, we repaired 2,312 consecutive hernias in 1,828 patients, 284 of which were recurrent. We present our results in terms of quality of repairs, recurrence rates (0.4%), morbidity rate (8%), and mortality rate (0.8%).

This technique involves the placement by an open incisional route of a large preperitoneal sheet of mesh for initial treatment of all inguinal hernias - including scrotal, giant or femoral - to ensure a definitive solid muscular wall, even for recurrent hernias.

Repair of an inguinal hernia, the second most common operation after appendectomy, is always a dilemma for surgeons, with various options available - the open and laparoscopic approaches, the use of different types of material and different situations (pre-muscular, retromuscular), size and fixation of a mesh.

In the past, only the recurrence rate was taken into account as the first goal in hernia repairs. Today, by laparoscopic or open routes, new challenges have to be faced to obtain not only a solid repair with low morbidity and mortality rates but also a painless postoperative period, a short hospital stay, an inexpensive technique, a technique easy to teach and feasibility of carrying out repairs by every surgeon.[1]. The incidence of recurrences after prosthetic methods is about 1% in large series published with a sufficient follow-up. The surgical community observes and has to accept higher recurrence rates (3–10%) for patients that do not require or are unsuitable for the use of mesh - such as children, young adults, emergency or septic patients - in whom a Shouldice technique or another kind of nonprosthetic repair is recommended.

We reported our last prospective study using our typical technique[2] for about 2,312 repairs, with a mean follow-up of 8 years and satisfying late results.

## MATERIALS AND METHODS

From January 1992 to December 2002, 2,312 hernias were operated on in our department. Of these, 284 hernias have been operated upon for recurrences. We excluded from our study young patients under 18, people with wall, cutaneous or septic problems and emergency cases with strangulated intestines.

The mean age for our patients was 56 years (range = 19–89). There were 242 females and 2,070 males. The types of primary hernias were indirect in 51%, direct in 32%, combined 5%, femoral 2% and bilateral 10%. The patients operated for recurrences were 238 men and 46 women; they had been operated before by Bassini (72 cases), Shouldice (22), McVay (48), 22 cases with a prosthesis, 11 after Lichtenstein, laparoscopic in 23 cases: TEP and TAPP techniques. In 78% of these cases, it was a first recurrence. In 85 cases, we did not recognize the first technique used. It was the second operation in 42 cases, the third in 22. In one case it was the seventh and one other case presented with a giant hernia.

The interval to recurrence was less than 6 months in 72 cases, between 6 and 12 months in 31 cases, between 1 and 5 years in 36 cases, more than 5 years in 36 cases and unknown in others. The patients had been operated under general anesthesia in 84%, rachi 10%, local 6%. Ambulatory surgery had not been performed in this period. The mean hospital stay was 3 and was gradually becoming shorter. All patients have been operated upon under anticoagulant drugs and antibiotic prophylaxis (cephalosporin).

### Technique

A short inguinal incision (7 cm) is made and the external oblique aponeurosis is incised according to its direction. A finger dissection is done, freeing a wide space between the aponeurosis and the internal oblique muscle, avoiding damage to the ilio-inguinal and ilio-hypogastric nerves. This preparation is always performed to allow subsequent tension-free closure of the Fruchaud foramen after placement of the prosthesis.

The spermatic cord is carefully identified and isolated. As in other open techniques, the sac is localized from the spermatic vessels; the place of the epigastric vessels is recognized. The posterior floor and the fascia transversalis are opened from the deep ring to the pubis and the inferior epigastric vessels are routinely divided distal to the funicular artery, once the patency of the internal iliac artery has been verified. This key-managing gives a wide access to the Bogros' preperitoneal space and particularly to the posterior aspect of the large muscles of the abdomen.[3]

**Figure 1:**
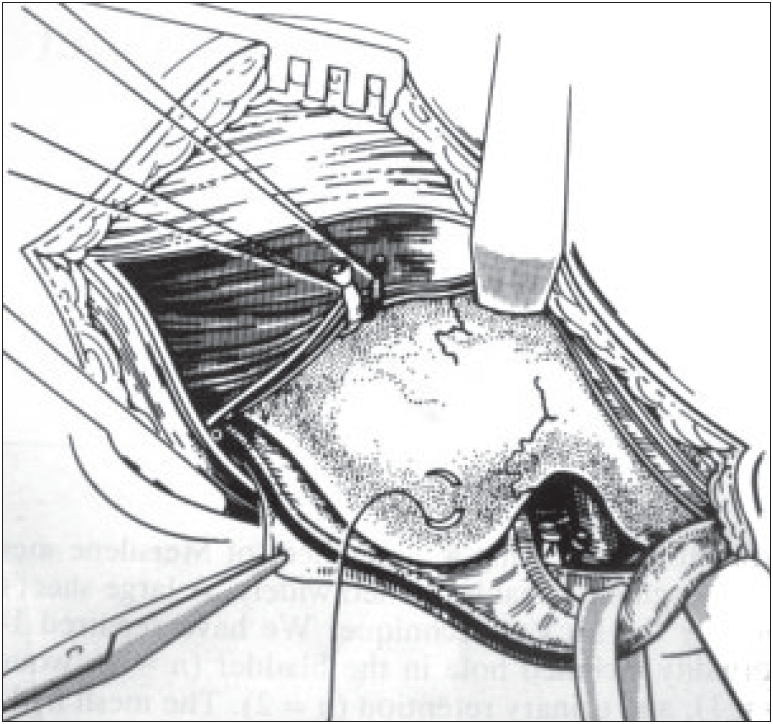
Exposure of the area after section on the fascia transversalis, division of epigastric vessels and displacement of the cord

The cremasteric muscles are divided, the cord is released at the internal ring and the different elements of the spermatic cord are separated from the peritoneum and dissected as deeply as possible towards the origin of the spermatic artery and the pelvic portion of the vas deferens to separate them. A triangular space appears between the spermatic artery and the vas deferens. The apex of the triangle is inguinal, while its base is pelvic. In this manner, the elements of the spermatic cord are well separated from the peritoneum and the preperitoneal fascia [Figure 1]. Cooper's ligament is then freed (or refreed) as are the structures located on the inner aspect of the obturator foramen. The superficial aspect of the anterior parietal peritoneum is freed by finger dissection while traction is applied to epigastric pedicle.

Once dissection is complete, the parietal portion of the peritoneum is entirely free from the iliac fossa and ample room has been provided to place a large prosthesis over it. The hernia sac (or sacs if indirect and direct) is then dissected and opened and resected only in case of a very large one. If not, it is routinely pushed back posteriorly.

A large unsplit piece of unabsorbable mesh 15 × 17 cm is then aseptically prepared. Usually we use a polyester one; we always choose a very thin and supple mesh. It is inserted below by passing it behind the inferior part of the peritoneum, descending beyond Cooper's ligament as far as the internal obturator and levator ani muscles. The mesh is spread out laterally and posteriorly over the cord structures, while the external vessels remain below and inserted between the peritoneum and these structures.

Because the cord is parietalized, no mesh-slit needs to be made for the spermatic cord as in Rives technique - it is our guarantee against any hole in favour of an eventual recurrence.

**Figure 2:**
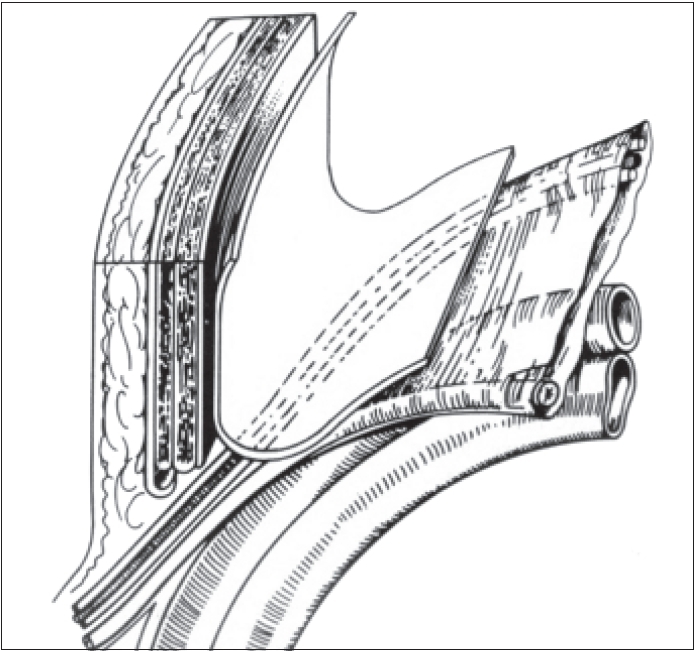
Position of the mesh over the peritoneum and above the parietalized spermatic cord

The mesh is then made to bend over on the peritoneum; the superior portion of the mesh is carefully positioned behind the fascia, the conjoint tendon, the transverse muscle and the rectus muscle - without any folds - reaching the midline and up to 10 cm above and behind the transverse muscle [Figure 2].

**Figure 3:**
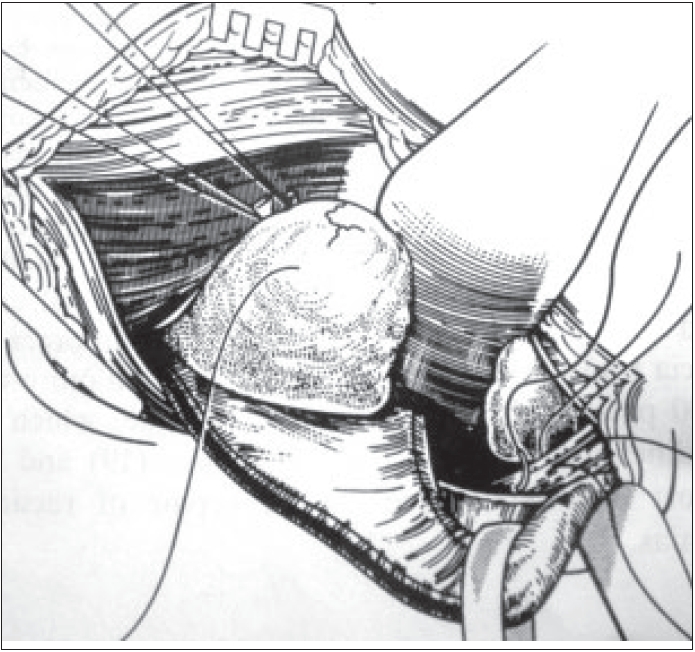
Dissection of the sac: nonabsorbable sutures passed through Cooper's ligament

**Figure 4:**
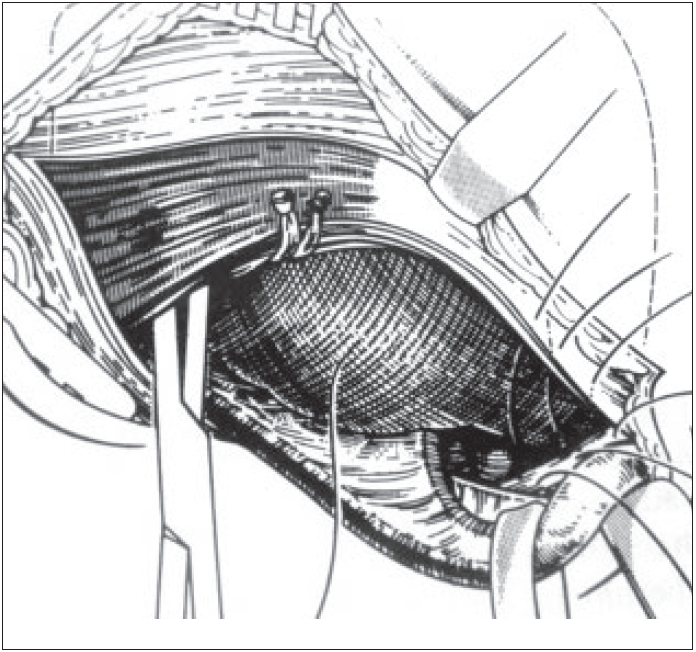
Closure of the wall over the prosthesis

A closure of the muscular foramen is done as in McVay technique but without any tension because of the wide dissection done between the external aponeurosis and the conjoint tendon. The most internal fibers of the transverse and internal oblique muscles are fixed on to the Cooper's ligament with three nonabsorbable sutures placed on the ligament before the insertion of the prosthesis [Figures 3 and 4]. The first internal suture fixes the internal part of the prosthesis. In case of any tension, a discharging vertical incision is made on the anterior pre-rectal aponeurosis.

The cord and all its elements, including the vas deferens, vessels and its preserved nerves are placed laterally to reconstitute a bayonet configuration. The external oblique fascia is closed in front of the cord Without excess up to manage a sufficient hole for the cord. The wound is then closed without drainage. This operation lasts about an hour.

## RESULTS

All the patients were seen 1 week, 1 month, 2 months after their operation. Ninety percent of them have been examined after 1 year, 83% after two years, 79% after 3 years.

The mean follow-up was 8 years. Because the percentage of lost patients was above 10%, the statistical method of maximal bias has been applied for the definitive results.

### Mortality (0,8%)

During the postoperative period 16 patients, with preexisting cardiac or pulmonary diseases in old age, expired due to myocardial infarctions, embolisms, pulmonary infections. Nine patients died after this period, due to other causes, with their hernia repairs intact.

### Morbidity

The global morbidity has been 8%. This includes the following:

Superficial hematoma - 3.4%

deep hematoma -0,6%

Urinary complications - 1.8%

Medical complications - 2.6%

Septic complications with prosthetic removal - 2%

Postoperative pain - 0.2%

Recurrence rate (maximal bias) - 0.4%

We have been obliged to reoperate several patients for deep hematomas imputed to heparin with a systematic replacement of the mesh. Because the mesh is only fixed on the internal part of the Cooper's ligament, it has been observed in such cases that the mesh has been often displaced. In order to avoid a recurrence, we decided to operate all these patients with large hematomas with evacuation to verify and to replace the mesh in good position.

In case of suspected deep infection, we removed the mesh in 2% of cases after waiting. In the majority of cases, after removal no recurrence occurred after a long follow-up.

## DISCUSSION

Multiple hernia-cure techniques are proposed to surgeons. Each one has his favorites. We proposed our technique in 1984; at that time, only nonprosthetic techniques, like Bassini and Shouldice procedures, were being performed. The laparoscopic era, since 1991, opened up large possibilities for the use of the prostheses.[4] During the same time, competition began between TEP and TAPP and between the superficial mesh technique of Lichtenstein[5] and the plug technique of Gilbert,[6] Robbins and Rutkow. Another competition appears with the making of numerous kind of prosthesis and the multiplication of the factories manufacturing new material.

It is not our goal to compare in this paper the advantages of all these cures or of these materials, but we intend only to emphasize the advantages of the technique we used. The first advantage we advocate is that this operation is able to cure all types of hernias: primary as well as recurrences.[7] We also advocate this technique because the deep dissection of Bogros' space identifies femoral hernias and any structures passing through the obturator orifice.[3] The concept of a large preperitoneal mesh overlapping the peritoneum has been outlined by Stoppa[8] after Rives.[9] But the so-called Stoppa operation by midline incision or transversal as Rignault appears more indicated for bilateral hernias. This operation is difficult to perform and its recurrence rate is high (6%). On the contrary, the placement of one prosthesis on one side - as we do by our technique - is easier to perform without folding and sliding of the material. Our method is easy to teach, because the inguinal route is known to a majority of surgeons, experts in anatomy. Our operation can be done if necessary under rachi anesthesia and local anesthesia. The position of the prosthesis avoids injury to the wound's nerves; they are often damaged in cure using a pre-muscular mesh (in Lichtenstein or Mesh plug procedures).

Laparoscopic procedures using the concept of a preperitoneal prosthesis, in good hands, give very good results,[10] but the placement of a mesh needs general anesthesia, specific expensive material (trocars, etc.) a lot of specific meshes and a long learning curve. At this time in France about 80% of surgeons are reverting to the open, because of some very rare but existing vital damages by laparoscopic surgery and an equivalent recurrence rate.

The only advantage pointed out by the laparoscopic supporters is less pain after operation than after pre-muscular mesh procedure and a possible ambulatory use.

In our technique, using new local analgesic products, our patients have a shorter hospital stay: actual mean-stay time - 1 day. The return to work is comparable with laparoscopic procedures.

## CONCLUSION

We have to insist upon the possible use of our technique in all types of hernias with all kinds and types of prostheses: the surgeon can cut the mesh as per the patient's anatomical measurements. We always used a very thin and soft prosthesis (polyester), which is not possible by laparoscopic procedures.

There is no need to fix the prosthesis (as in Lichtenstein or plug-mesh procedures), nor any need to use fibrin or other types of expensive glues.[11–13] The classic danger of an infection is very low in this technique when usual rules of asepsis are carefully respected.

All recurrences can be operated upon by the same technique, even when a prosthetic material has been placed laparoscopically in a previous operation.

This operation is indicated in all types of hernias; it is recommended to teach the surgeons they need only one preferred reference.[14]
